# A method for unsupervised learning of coherent spatiotemporal patterns in multiscale data

**DOI:** 10.1073/pnas.2415786122

**Published:** 2025-02-14

**Authors:** Karl Lapo, Sara M. Ichinaga, J. Nathan Kutz

**Affiliations:** ^a^Department of Atmospheric and Cryospheric Sciences, University of Innsbruck, Innsbruck 6020, Austria; ^b^Department of Applied Mathematics, University of Washington, Seattle, WA 98195; ^c^Department of Electrical and Computer Engineering, University of Washington, Seattle, WA 98195

**Keywords:** multiscale, complex systems, dynamic mode decomposition, unsupervised learning, data-driven modeling discovery

## Abstract

The unsupervised and principled diagnosis of multiscale data is a fundamental obstacle in modern scientific problems from, for instance, weather and climate prediction, neurology, epidemiology, and turbulence. Multiscale data are characterized by a combination of processes acting along multiple dimensions simultaneously, spatiotemporal scales across orders of magnitude, nonstationarity, and/or invariances such as translation and rotation. Existing methods are not well-suited to multiscale data, usually requiring supervised strategies such as human intervention, extensive tuning, or selection of ideal time periods. We present the multiresolution coherent spatio-temporal scale separation (mrCOSTS), a hierarchical and automated algorithm for the diagnosis of coherent patterns or modes in multiscale data. mrCOSTS is a variant of dynamic mode decomposition which decomposes data into bands of spatial patterns with shared time dynamics, thereby providing a robust method for analyzing multiscale data. It requires no training but instead takes advantage of the hierarchical nature of multiscale systems. We demonstrate mrCOSTS using complex multiscale datasets that are canonically difficult to analyze: 1) climate patterns of sea surface temperature, 2) electrophysiological observations of neural signals of the motor cortex, and 3) horizontal wind in the mountain boundary layer. With mrCOSTS, we trivially retrieve complex dynamics that were previously difficult to resolve while additionally extracting hitherto unknown patterns of activity embedded in the dynamics, allowing for advancing the understanding of these fields of study. This method is an important advancement for addressing the multiscale data which characterize many of the grand challenges in science and engineering.

## Significance

Multiscale data remain exceptionally difficult to evaluate and characterize even for fields for which we have well-established physical laws or understanding. Modern grand challenge scientific problems from weather and climate, neuroscience, biological systems, finance, etc., all inherently exhibit high-dimensional, multiscale dynamics, which are those that occur across orders of magnitude, with overlapping scales, nonstationarities, and/or translating features. Despite advances in modern machine learning techniques, principled approaches for unsupervised extraction of features remain fairly intractable. We present the multiresolution coherent spatio-temporal scale separation, which overcomes many of these challenges with a hierarchical, unsupervised approach to extracting patterns from multiscale data. We demonstrate the method on three real-world multiscale systems, trivially retrieving known patterns while extracting otherwise hard to identify patterns.

From electromagnetism and quantum mechanics to fluid dynamics, the observed patterns in nature have been critical for hypothesis formulation and testing (1). Data has enabled the discovery and construction of the physical laws of nature, as well as uniscale descriptions at specific levels of temporal and spatial resolution (e.g., Schrödinger equation for quantum mechanics at angstrom scale, Maxwell equations at hundreds of nanometers for visible light, etc.). The problem in modern science, especially in grand challenge science and engineering settings (2) are related to deducing patterns in the complexities of multiscale data, where orders of magnitude of temporal and spatial scales are jointly measured and observed. Thus, there is no unifying uniscale model description, rather dynamics are driven by different processes at, for instance, micro-, meso-, and macroscales, all of which are coupled together in ways that are poorly understood. Instead, there is a general scale-separation problem in which we cannot even properly identify the patterns of activity at each scale in order to generate and test hypotheses about the underlying dynamics and their couplings.

A multiscale diagnostic is therefore critical for advancing and testing hypotheses about the underlying physical laws and their couplings in the grand challenges in science and engineering such as weather and climate modeling, neuroscience, biological systems, finance, etc., all of which inherently exhibit high-dimensional, multiscale dynamics (2). The interplay between deductive and inductive reasoning for scientific theories has always required clear observations of a process in order to advance a successful theory. It is simpler to explain patterns once it is understood what you are looking for (e.g., supervised learning), but when the existence of a pattern is undetected and unknown, the underlying dynamic process remains a latent contribution and true understanding is incomplete. This is why unsupervised algorithms for finding patterns are of such exceptional value: they fundamentally can help to accelerate the process of scientific understanding by the discovery of patterns hidden to observers. Multiscale dynamics are perhaps the most difficult from which to extract patterns, as the significant interactions across temporal and spatial scales prevent a clear observation of the underlying processes. These complexities have likely contributed to a significant lack of algorithmic development and analysis tools for multiscale phenomenon. Demonstrated here is an algorithm capable of extracting hitherto unobserved patterns of activity in exceptionally challenging multiscale problems.

Partially as a response to the challenges of multiscale dynamics, machine learning has emerged as a leading method for analysis. However, machine learning has clear drawbacks in terms of computational costs (3) and interpretability (4–6). Thus, there is a clear need for unsupervised methods which automate the extraction of patterns and diagnoses of multiscale data. Data-driven model discovery has emerged as powerful paradigm for handling data with complex dynamics by attempting to discover missing dynamics from data directly instead of from first principles (7). These approaches are powerful even in fields for which the first principles are known, as behavior at a larger scale tends to emerge from complex interactions at smaller scales, such as is the case for fluids (8, 9), turbulence (10), weather (11–13), neurology (14, 15), and climate (16–19), among many others. However, real multiscale datasets remain a challenge for data-driven model discovery.

It is often stated that data are high-dimensional or multiscale, but these definitions are in general used loosely. Here, we explicitly define multiscale dynamics as those characterized by a combination of at least two of the following properties: being multivariate (i.e., acting along multiple dimensions simultaneously), containing process scales across orders of magnitude, being nonstationary, or having invariances such as translation and rotation. Additionally, these data often contain noise or uncertainty, i.e., from instrument error. We call data characterized by multiscale dynamics “multiscale data.” Data with these properties are precisely those which are least amenable to analysis since no method can work with all of the aforementioned properties of multiscale data. For instance, multiresolution analysis (MRA) can provide multiscale information on the dynamics along a single dimension (e.g., time or along a spatial dimension) but not multiple dimensions simultaneously (1). Modal analysis can reveal dominant spatial patterns (8, 9) but these methods cannot robustly handle multivariate data (e.g., ref. 20), nonstationary (e.g., ref. 21), and/or translating processes (22). As such, diagnosing multiscale processes, especially in an unsupervised approach with minimal tuning, is a fundamental methodological need for a diverse set of disciplines. Consequently, many of the grand challenges in science and engineering are, in essence, challenges of multiscale data (2).

**Fig. 1.**
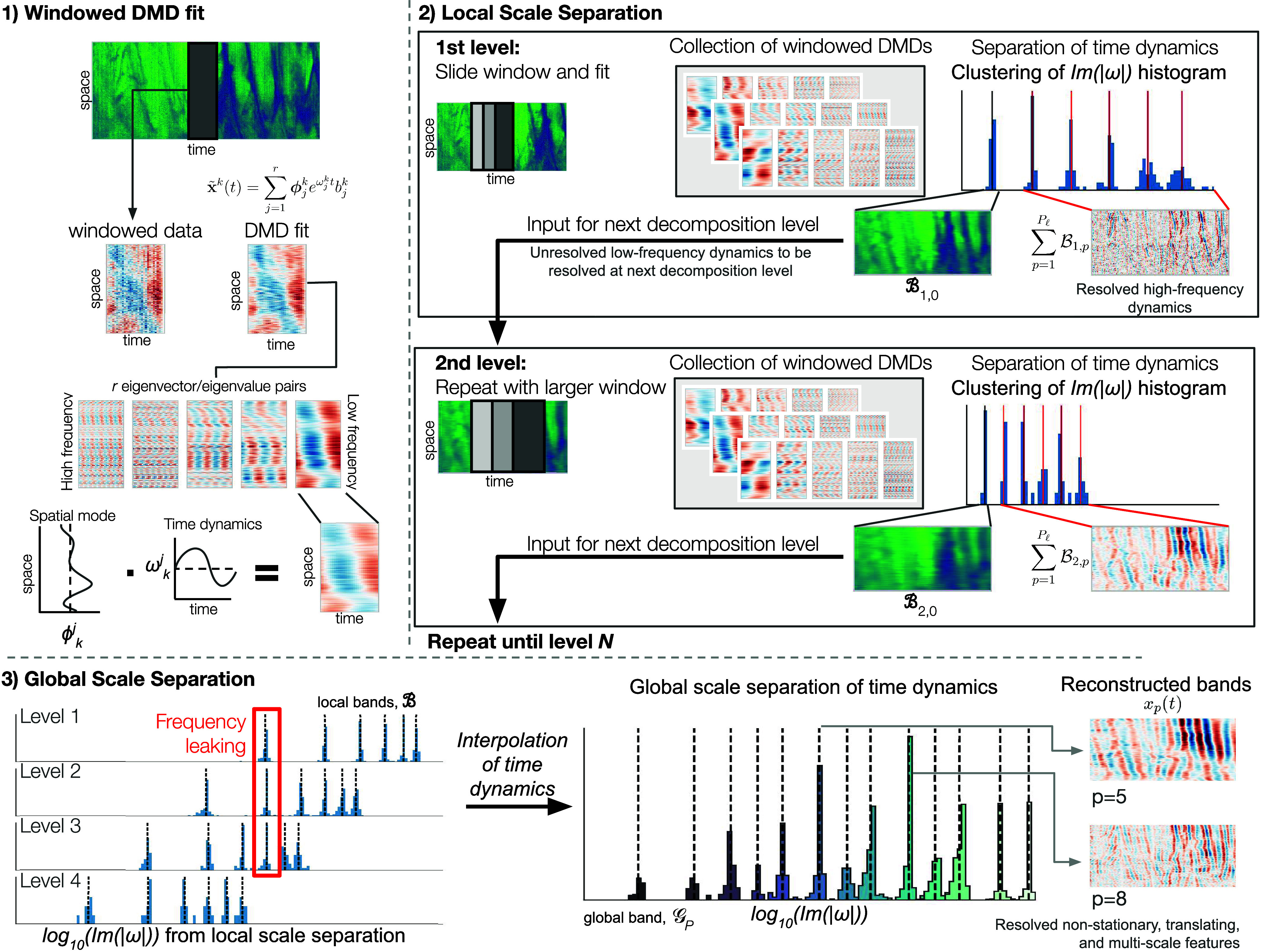
An overview of the mrCOSTS algorithm. (1) The data are windowed and a DMD model is fit to each. The resulting fit is composed of several time dynamics with distinct frequencies with a corresponding spatial mode, which can take on any functional shape. For the local scale separation (2), the window is slid across the data resulting in a collection of DMD fits. The time dynamics are clustered yielding bands composed of eigenvalue/eigenvector pairs which share time dynamics. The bands with the highest frequency are removed and the data reconstruction using the lowest frequency band is given to the next decomposition level. At the next level the window size is increased, allowing slower time dynamics to be fit, and the process is repeated. (3) Finally, the collection of frequency components from the local scale separation need to be merged to account for frequency leaking prior to a final, global scale separation. The resulting bands decompose the complex multiscale physics of the input signal.

To this end, we present multiresolution coherent spatiotemporal scale-separation (mrCOSTS). It is specifically tailored for robust, interpretable diagnoses of multiscale dynamics; a hierarchy of scales are robustly identified in an unsupervised fashion with minimal tuning. In short, mrCOSTS operates on a principle similar to the continuous wavelet transforms. A sliding window is applied to the data and in each window a Dynamic Mode Decomposition (DMD) model is fit (Part 1, Fig. 1). DMD has the desirable property of describing coherent spatiotemporal modes. Each mode is interpreted as a coherent spatial mode governed by a single set of temporal dynamics and a small number of coherent modes can robustly reconstruct complex dynamics (Part 1, Fig. 1). After fitting each window, we recover a collection of DMD models describing a range spatiotemporal dynamics (Part 2, Fig. 1). The temporal dynamics of the DMD models are clustered into bands. The high-frequency components are well resolved and removed. The low-frequency component is not well-resolved and is withheld as the input for the next decomposition level with a larger window, which can now resolve more of the low-frequency component of the signal. Finally, as information tends to leak between levels, a final global determination of the temporal bands is made, yielding an interpretable decomposition capable of resolving nonstationary, transient, multiscale, and high-dimensional features (Part 3, Fig. 1).

To demonstrate the ability of mrCOSTS, we diagnose three systems characterized by complex multiscale dynamics. We choose to demonstrate mrCOSTS on real systems instead of toy models as the data from toy models do not approximate the true complexities of multiscale data. That said, mrCOSTS was developed and tested against toy models and an example of mrCOSTS diagnosing a multiscale toy model can be found as part of the PyDMD tutorial of mrCOSTS (23). In the first example, we diagnose patterns of sea surface temperature (SST) over the central Pacific, focusing on the 2015–2016 extreme SST anomaly. In the second example, we examine Local Field Potential (LFP) observations of a monkey’s motor cortex during a trained task. Finally, we decompose ground-based remote sensing retrievals of horizontal wind speed in the mountain boundary layer (MoBL).

## mrCOSTS

mrCOSTS builds on the principles of multiresolution dynamic mode decomposition (mrDMD; ref. 22), specifically the sliding mrDMD method (24), but is tailored to the complexities of multiscale data.

DMD is a method for data-driven model discovery (1, 10, 25) that provides a low-rank, interpretable model of coherent spatiotemporal features. However, a basic assumption in many variants of the DMD (for a list of commonly applied variants see ref. 23) is that the data can be approximated by a (relatively) small number of coherent spatiotemporal modes. For real-world systems, especially those characterized by multiscale dynamics, this assumption on the time dynamics is not often valid. Consequently, DMD analysis relies on strategies such as omitting non-steady-state periods (9, 21, 26). In response, the multiresolution DMD (mrDMD) was developed (22, 24), however, we found these methods, while sufficient for synthetic test data, struggled with real multiscale data leading to the development of mrCOSTS.

The mrDMD framework is given by Eq. **1**, in which input snapshots $x\left(t\right)\in R^{n}$ collected across times ${\left\{t_{i}\right\}}_{i=1}^{m}$ are sequentially windowed and fit with a DMD model so that

[1] $$\begin{array}{c} {\tilde{x}}^{k}\left(t\right)=\sum_{j=1}^{r}\phi_{j}^{k}e^{\omega_{j}^{k}t}b_{j}^{k}+c_{k}. \end{array}$$

We use ${\tilde{x}}^{k}\left(t\right)$ to indicate an approximation to the windowed data, $x^{k}\left(t\right)$. We use k to index the data windows so that snapshots belonging to the kth window, denoted as $x^{k}\left(t\right)$, are approximated by the decomposition given by Eq. **1**.

The input data can have arbitrary dimensionality but the non-time dimensions must be flattened to a single spatial dimension, similar to the data shape requirement for most machine learning algorithms (27). Similarly, x(t) may be composed of multiple variables, as demonstrated in the MoBL example. In the case of multiple variables, the DMD model finds the coherent spatiotemporal patterns shared among the variables.

Prior to decomposition, each window is normalized by removing the time mean, denoted $c_{k}$, which must be added back in to reconstruct $x^{k}\left(t\right)$ (28). We use j to index the DMD eigenvalue (ω)/eigenvector (ϕ) pairs up to rank r, where b gives the amplitude of the pair. The spatial structure of the data, which is encoded by ϕ, has time dynamics that are determined by ω, where the real component of ω governs exponential growth or decay and the imaginary component governs oscillatory behaviors (Part 1, Fig. 1). Eq. **1** is then fit to sliding windows of the data with a fixed window length in time, which constitutes a decomposition level. Thus for a given decomposition level, we obtain a collection of DMD fits that describe the coherent spatiotemporal structures of the data (Part 2, Fig. 1).

Since the decomposition given by Eq. **1** differs across all windows k for a given decomposition level, indexed with ℓ, we cluster all $Im(|\omega_{j}^{k}|)$ values from decomposition level ℓ. This yields clustered eigenvalue/eigenvector bands, denoted B, that are local to the ℓth decomposition level (Part 2, Fig. 1). $B_{\ell ,0}$ has the smallest Im(|ω|), i.e., the slowest frequencies, and is characterized by time scales that are longer than the window length. This band is usually decomposed into more refined time scales at larger decomposition levels and consequently $B_{\ell ,0}$ is not well-resolved. In contrast, the bands with larger Im(|ω|), i.e., higher frequencies, which we denote in order of increasing frequency using $B_{\ell ,1},B_{\ell ,2},\cdots ,B_{\ell ,P_{\ell }}$, are better resolved at the ℓth decomposition level. The contribution of any band $B_{\ell ,p}$ to the signal can be recovered by

[2] $$\begin{array}{c} {\tilde{x}}_{\ell ,p}\left(t\right)=\sum_{k}\sum_{j\in B_{\ell ,p}}\phi_{j}^{k}e^{\omega_{j}^{k}t}b_{j}^{k}+c_{k}. \end{array}$$

We thus use Eq. **2** for p=0 to reconstruct the unresolved low-frequency components of the data at decomposition level ℓ so that it may be used as input for the next decomposition level. The background value, $c_{k}$, is only included for p=0 and is otherwise excluded.

The algorithm is then repeated for decomposition level ℓ+1, which uses a larger data window with a length selected by the user (*Hyperparameters*). In doing so, high-frequency components which are resolvable by DMD at decomposition level ℓ are removed, while the low-frequency components are retained for further scale separation at larger window sizes. The algorithm is repeated until the largest desired decomposition level, N, is reached.

The general algorithm outlined above, which follows the broad outline of the mrDMD (24), is called the local scale separation. A normalizing factor was omitted for reconstructing the overlapping windows for brevity. The windowed DMD fit is most robust near the center of the window and less reliable near the edges (24). Overlapping DMD windows are strongly weighted toward the center of the window as a consequence of the poor fitting at window edges. For this reason, mrCOSTS is least reliable at the edges of the time domain. This error is analogous to the cone-of-influence from continuous wavelet transformations (29) and is thus most noticeable at the largest decomposition level with the longest time scales.

Performing this algorithm recursively on data without clearly separable time dynamics, i.e., multiscale data, can lead to poor results due to the leaking of information across decomposition levels (part 3 in Fig. 1) as well as other deficiencies in the original algorithm (*Methods*). To remedy the leaking of frequency components between levels, a global scale separation has to be performed on the fast frequencies from all decomposition levels. For this step, the ω components from all of the local frequency bands $B_{\ell ,p}$ for all ℓ and p>0 are interpolated to the mean time of $B_{0,p}$’s windows using a nearest neighbor approach. The interpolated ω are then used to perform the global scale separation and are then reindexed back to the original decomposition window’s time windows. The resulting globally scale separated bands, $G_{0},G_{1},\cdots ,G_{P}$, listed in order of increasing band frequency, are a complete scale separation that includes the leaked component between each level. We again use p to index the globally scale separated bands.

We can then reconstruct the contribution of $G_{p}$ to ${\tilde{x}}_{p}\left(t\right)$ using

[3] $$\begin{array}{c} {\tilde{x}}_{p}\left(t\right)=\sum_{k}\sum_{\left(j,\ell \right)\in G_{p}}\phi_{j,\ell }^{k}e^{\omega_{j,\ell }^{k}t}b_{j,\ell }^{k}. \end{array}$$

A global reconstruction of the mrCOSTS fit can be found by

[4] $$\begin{array}{c} \tilde{x}\left(t\right)\approx \sum_{p}{\tilde{x}}_{p}\left(t\right)+c_{P,k}. \end{array}$$

The background value of each window in $G_{0}$ (the lowest frequency band), $c_{0,k}$, needs to be included for the global reconstruction of the signal. Note, $G_{0}$ is defined as the low-frequency component of the largest decomposition level and therefore may not contain well-resolved dynamics. The influence of multiple selected bands can be found through Eq. **4** by simply summing over the desired p and omitting the background value. For ease of interpretability, we identify G bands according to the cluster centroid period or frequency (vertical dashed lines in part 3, Fig. 1), depending on which is more intuitive for the system of interest.

A useful property of mrCOSTS is that each $G_{p}$ for p>0 is an approximation of the contribution by coherent spatiotemporal processes with a narrow frequency range to the fluctuating component of Reynolds decomposition. This property makes mrCOSTS especially powerful for the decomposition of systems governed by the Navier–Stokes equations as well as other systems with similar multiscale hierarchies. Another important property is that nonspatially coherent processes, such as white noise, cannot be fit by mrCOSTS as white noise is not spatially coherent with an oscillatory time scale. As a result, $\tilde{x}$ is an approximation of the denoised x.

### Distinction from Other Methods.

Other methods have various limitations when applied to multiscale data. Modal analyses, such as principal component analysis (PCA), breaks the correlation between spatial modes and time dynamics, an undesirable effect that limits the diagnoses from PCA (10, 30) and can even lead to misleading results (20). Modal analysis, which includes most variants of DMD, also represents invariances like a traveling wave using a large number of modes that are poor representations of the underlying physics (1, 8, 9, 20, 22, 31, 32). Further, these methods often require a large number of modes to account for processes across order of magnitude time scales (1, 31). Time-frequency analysis (33–36) which represents the frequency information in a time signal, such as Continuous Wavelet Transforms and power spectra, can account for nonstationary processes across process scales, but can only diagnose these processes along a single dimension (typically time) but not multiple dimensions simultaneously (1, 32, 33, 37, 38). It is increasingly common to predict multiscale data using machine learning models (i.e., neural networks). Although machine learning models are capable of handling multiscale features, like translations, the success of the model is based upon supervised learning where labeled data must be provided. To date, unsupervised machine learning techniques have not proven successful in automating the extraction of features for building physically interpretable models (5, 6). *SI Appendix*, Table S1 contains a summary of commonly used unsupervised methods’ ability to diagnose multiscale data.

In specific regards to DMD methods, DMD struggles with diagnosing transient or translating processes, as these processes cannot be globally represented using a single exponential time dynamic (9, 21, 22, 26). Physics-informed DMD (PiDMD) can account for some multiscale features, but requires a priori knowledge of the underlying physics (39), which is problematic for an unsupervised method. These drawback can be overcome using windowed approaches. The nonstationary DMD (ns-DMD; *SI Appendix*, Table S1, ref. 40) can diagnose intermittent or transient phenomenon but was not constructed to separate time dynamics with scales across orders of magnitude. The mrDMD algorithm performed well on synthetic data (i.e., refs. 22 and 24), however, the algorithm did not provide robust diagnostics of real multiscale data, such as presented in this manuscript. To address the shortcomings with mrCOSTS, substantial modifications to the sliding mrDMD were made such as the addition of the global scale separation (Part 3, Fig. 1) and eigenvalue constraints (see *Algorithm* details). Thus, the ability of mrCOSTS to diagnose these real-world multiscale data is a substantial methodological distinction.

## Results

Each example is characteristic of the multiscale dynamics which generally frustrate other analysis methods and are presented in order of our subjective assessment of complexity as well as unknown dynamics. The SST example provides a validation of sorts due to the well-known time scales governing the system. Using mrCOSTS, we recover these known time scales while also recovering the generally poorly described spatial patterns as well as previously neglected time scales critical to the description of this system. In the neurology example, we trivially isolate and characterize well-known frequency bands and in doing so isolate the previously poorly described translating spatial patterns. Finally, in the MoBL example, we recover dynamics not previously characterized for a system that generally frustrated objective analysis.

### Sea Surface Temperature.

Climatic SST patterns of the Pacific Ocean, especially the El Ni$\tilde{n}$o-Southern Oscillation, are major modes of variability in the global atmospheric circulation, driving processes in the earth system with substantial societal impacts (41, 42). Predicting and understanding the dynamics of ENSO is a major research challenge (43, 44), especially in the context of possible changes to this mode of internal variability with climate change (42, 45).

ENSO is thought to have a dominant period between 2 and 7 y (36, 46) with a chaotic or complex quasiperiodic behavior (47). A wide variety of metrics for ENSO have to be used for various practical and research reasons (44, 48–53). To describe the mrCOSTS decomposition of SST, we use the Niño 3.4 box (48, 49) but perform the decomposition on a much larger region. ENSO is commonly described using monthly SST anomalies, which we denote as SST^∗^ (*Methods*).

**Fig. 2.**
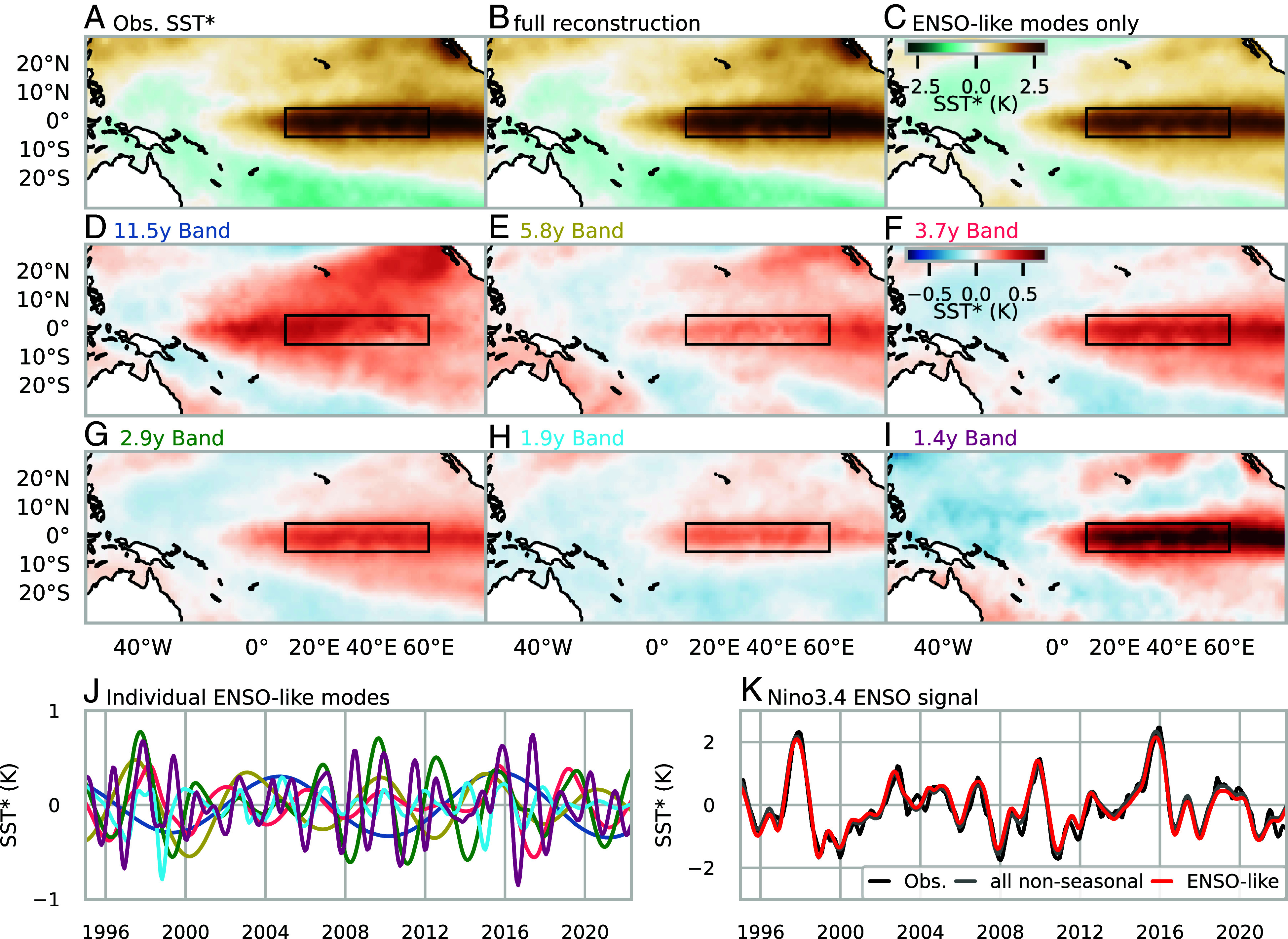
The mrCOSTS diagnosis of the ENSO anomaly during the period December 2015 to February 2016. These dynamics were recovered with no hyperparameter tuning. Shown are the (*A*) observed SST^∗^, (*B*) mrCOSTS reconstruction of SST^∗^ using all modes, and (*C*) mrCOSTS reconstruction using only the ENSO-like modes. (*D*–*I*) Each of the ENSO-like modes is reconstructed individually with the band centroid indicated in the subplot label. The Niño 3.4 box is drawn for reference. (*J*) The spatial mean of each of the ENSO-like modes inside the Niño 3.4 box are shown, yielding a time series of each mode’s contribution to SST^∗^. Each mode is color coordinated with the subplot titles in (*D*–*I*). (*K*) The time series of the observed, ENSO-like modes, and all nonseasonal modes SST* within Niño 3.4 are compared. The complex time evolution of ENSO is recovered and explained in no small part due to discovering the previously undetected time dynamics of the 11, 1.9, and 1.4 y bands.

To demonstrate the unsupervised diagnostic power of mrCOSTS, no hyperparameter tuning was performed. The mrCOSTS fit recovers the complex time dynamics of the SST observations, especially those at time scales longer than the annual cycle including the time scales commonly associated with ESNO (*SI Appendix*, Fig. S1). The resulting $G_{p}$ were visually inspected to identify a set of six bands with ENSO-like spatial patterns. The time scales of these modes varied from 1.4 to 11 y. Using these bands, the extreme ENSO anomaly in the period of DJF 2015–2016 (44, 51) was reconstructed using Eq. **4** (Fig. 2*B*) and Eq. **3** for the ENSO-like bands alone (Fig. 2*C*). The mrCOSTS decomposition accurately reconstructs the domain-wide SST^∗^ (Fig. 2 *A* and *B*) with most of the variability within the domain being reconstructed by the ENSO-like bands (Fig. 2*A*–*C*). The ENSO-like bands almost entirely decompose the SST^∗^ signal in the Niño 3.4 box with the reconstruction using all supraseasonal bands barely altering the reconstruction (Fig. 2*K*).

The six ENSO-like bands include three bands outside the time scales normally associated with ENSO: 11, 1.9, and 1.4 y. The 2015 anomaly was the result of all 6 ENSO-like bands having a positive anomaly at the same time (Fig. 2*J*), a remarkably rare occurrence in the reconstruction, not occurring for any other positive ENSO event. The largest contributor to the 2015 anomaly was the 1.4 y band, a time scale neglected in the characterization of ENSO, potentially connected to the known but difficult to characterize relationship between extreme ENSO events and the seasonal cycle (44). Other strong positive ENSO anomalies only had contributions from four or five of the ENSO-like bands, partially explaining either their weaker expression in the Niño 3.4 box or differences in spatial patterns and temporal evolution relative to the 2015–2016 event (44, 50, 51). For instance, the positive SST^∗^ in 1997 was a result of all ENSO modes in the positive phase except for the 11.5 y band (Fig. 2*J*). Further, the distinct ENSO-like modes are remarkably well-separated given that mrCOSTS was fit over the entire central Pacific Ocean over a 150 y long period. Previous analyses could not use the entire dataset to trivially recover these results but would need to do either some spatial aggregation (e.g., ref. 36) or careful data selection (e.g., ref. 48).

Unsurprisingly, given the known complexity and quasiperiodic nature of the ENSO signal, the individual ENSO-like bands go through periods of varying activity and do not exhibit clean oscillatory patterns. The 11 y band turns on in approximately 1980 but becomes regular afterward (dark blue line, Fig. 2*J*). In contrast, the 1.9 and 1.4 y bands exhibit little regularity (light blue and purple lines, Fig. 2*J*). Despite these nonstationary processes mrCOSTS was able to characterize and trivially diagnose the chaotic behavior of ENSO, simultaneously recovering the time dynamics and their associated coherent spatial patterns. While we recovered the dynamics known to exist in this system, we additionally identified three frequency bands which were otherwise not recognized for describing the system. Using these results we highlight how mrCOSTS can be used to diagnose in an unsupervised fashion known complex multiscale dynamics while also explaining dynamics that were previously difficult to characterize.

### Neurology.

Electrophysiological observations of the brain are thought to be a method for uncovering the basis of cognition in neural processes (14). The high-frequency component of the signal (>500 Hz) is related to the spiking of individual neurons near the electrode while the low-frequency component of the signal (<500 Hz; the local field potential, or LFP) offers an insight into the integrated behavior of the entire neural circuit, albeit with substantial complications to this general interpretation (15).

The relationship between LFP signals and specific neural pathways is complex, as the contributions to the LFP signal from specific neural circuits are ambiguous and are composed of complex spatiotemporal signals (14, 15). Temporal dynamics occur across a range of scales and are activated by different spatial regions/neural circuits and sometimes even the same set of neurons. Consequently, the “spatial factors [of LFPs] are among the most important issues to be explored in depth in the following years” (15). Developing mathematical tools for analyzing the LFP is hence seen as one of the major needs in neuroscience (14, 54). DMD has been found as a promising approach for analyzing LFP data (40, 55), but the multiscale nature of these data frustrates the application of DMD more generally. From this perspective, we therefore reframe the problem of interpreting LFPs as a challenge, in large part, in interpreting data with multiscale dynamics.

The LFP signal is often described in terms of frequency bands, making the scale-separation task particularly amenable to decomposition with mrCOSTS. The observations we use cover frequencies describing contingent negative variation (CNV) related to delayed tasks (<2 Hz; ref. 56), the movement-related potential (MRP) in the motor cortical areas which encodes movement type and direction (3 to 15 Hz; refs. 57 and 58), beta waves (15 to 30 Hz; refs. 59 and 60), and gamma waves(>30 Hz; ref. 59). The MRP is of particular interest due to its relationship with specific movements through complex spatiotemporal patterns (54, 57, 61, 62). This relationship is particularly difficult to describe, yet highly desirable to understand for applications such as neuroprostheses (e.g., ref. 62). Often the MRP is interpreted through intuitive transformations such as the aggregating a single electrode across multiple trials or aggregating the spatial components, neglecting aspects of the multiscale dynamics.

We use electrophysiological observations of a macaque’s motor cortex during a trained exercise (54). A monkey, with chronically implanted 10-by-10 Utah electrode array, was trained for a delayed reach-to-grasp task consisting of a cue for the type of grip (side or precision), a wait interval, and a go signal encoding how hard to grip (low- or high-force). Upon successful completion, a reward was dispensed. An advantage of these data is that they were released for the explicit purpose of developing analytical techniques for electrophysiological observations (63).

**Fig. 3.**
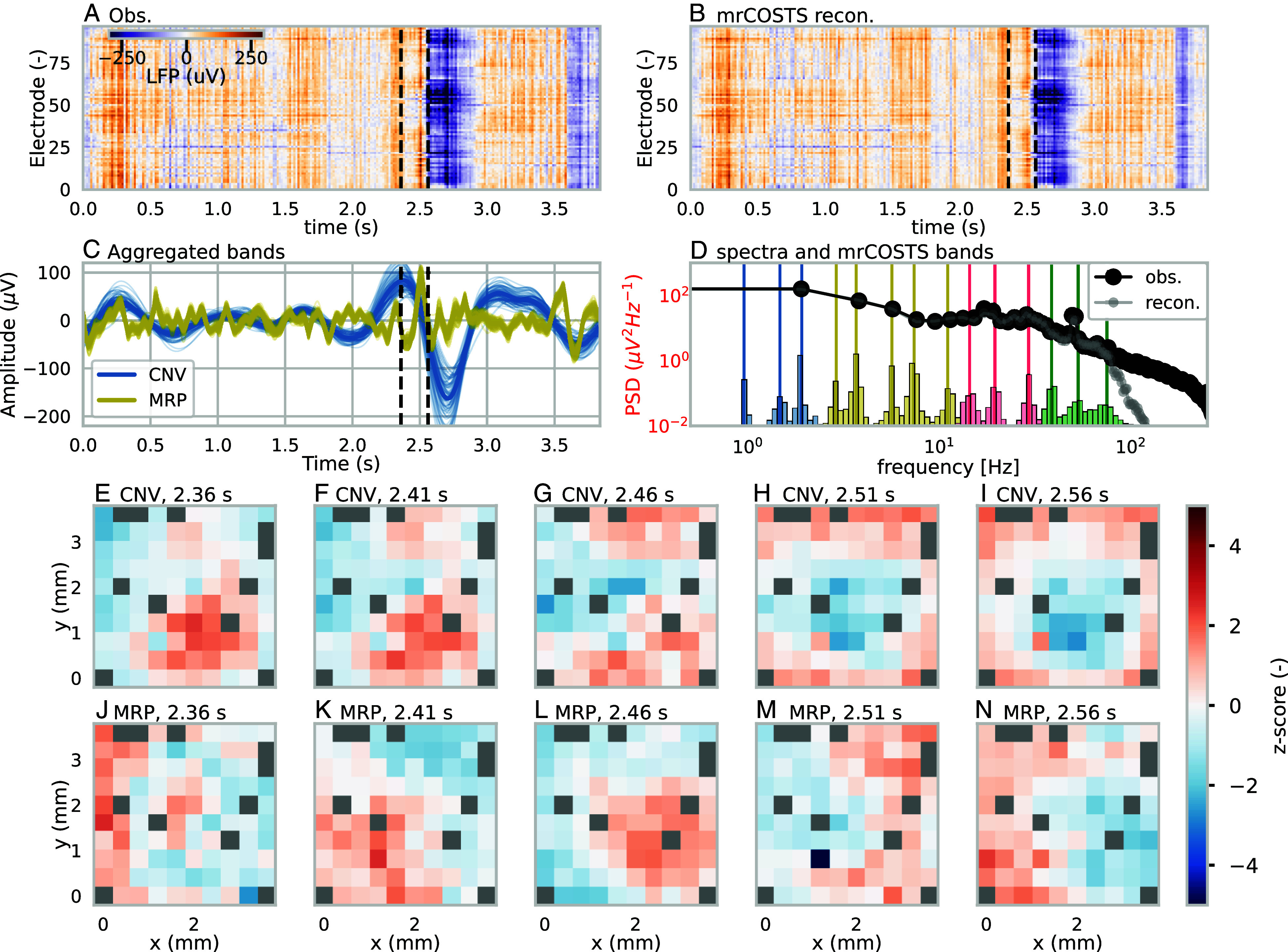
Decomposition of the LFP signal to the CNV and MRP bands using mrCOSTS for a selection of times. See Movie S1 for all bands and times. (*A*) Observed LFP and the (*B*) mrCOSTS reconstruction. (*C*) The aggregated bands were constructed to be consistent with the CNV (blue) and MRP (yellow) bands using Eq. **3**. These bands also shared characteristics during the movement. Each electrode from (*A*) and (*B*) is plotted as a separate line in (*C*). In (*A*–*C*), the first vertical dashed line denotes the cue for movement ($t_{1}$ = 2.36 s) while the second line indicates the beginning of the movement ($t_{2}$ = 2.51 s). (*D*) The mean power spectral density of the LFP (black line) and the mrCOSTS reconstruction (gray line). Plotted underneath is the histogram of the globally interpolated ω on a relative scale. The histogram is color coordinated with the assigned bands shown in (*C*) for the CNV and MRP and Movie S1 for all bands. The cluster centroids are indicated as vertical lines. The CNV (*E*–*I*) and MRP (*J*–*N*) bands are spatially reconstructed for five times from $t_{1}$ to $t_{2}$ with an even time spacing. The spatial reconstructions are standardized by a z-score to facilitate their plotting on the same color scale. Gray points indicate no data. The translating spatial patterns of these bands demonstrates how mrCOSTS can decompose these types of complex spatiotemporal signals, which are otherwise not amenable to analysis.

We applied mrCOSTS to trial two from monkey L (a side-grip low-force trial). The original hyperparameters successfully decomposed the system, but we found that a very low-frequency oscillation (≈1 s) was missed by the initial set of hyperparameters. Consequently, the hyperparameters were adjusted exactly once to include an additional decomposition level with a very large window size explicitly to capture this time scale. These very-low-frequency features are difficult to characterize a priori using typical methods like the power spectral density due to their poor resolution for very long time scales (Fig. 3*D*). However, mrCOSTS easily identifies the slower time dynamics of the system, demonstrating some of the diagnostic power of mrCOSTS over traditional techniques (compare the histogram of ω and band centroids to the power spectral density in Fig. 3*D*).

The reconstruction using the mrCOSTS fit captures all of the salient features of the observations (Fig. 3*A*, *B*, and *D*). A total of 14 bands were identified in the global scale separation (Fig. 3*D*). We aggregated the mrCOSTS $G_{p}$ into four bands using Eq. **3** to align with the CNV, MRP, beta oscillations, and gamma oscillations. All bands and their spatial structure are shown in Movie S1, whereas only the MRP and CNV are presented in Fig. 3 for visual clarity. The mrCOSTS fit recovers the spectra for the time scales slower than ≈75 Hz, meaning the dynamics of all of the neurological bands, especially the MRP and CNV, are well resolved (Fig. 3*D*).

With mrCOSTS, we trivially retrieve the spatial patterns of the MRP and CNV individually, which have distinct behavior during the grasping movement and reveal complex traveling spatiotemporal patterns (Movie S1 and Fig. 3*E*–*N*). The CNV aggregated band (blue line Fig. 3*C*) has a strong positive anomaly in the lower right hand corner of the electrode array (Fig. 3*E*) which travels out of frame to the bottom right corner and is replaced with a strong negative anomaly in roughly the same location (Fig. 3*E*–*I* and Movie S1). The MRP aggregated band also features translating processes. The positive anomaly in the lower left-hand side of the array travels to the right across the array (Fig. 3*J*–*N* and Movie S1). The MRP and CNV signals are not spatially static oscillations and appear to be characterized by these types of spatially translating features (Movie S1).

These sort of translating patterns of the MRP and CNV and their potential relationship with specific movements or events, respectively, are not well-explored even though the spatial patterns are known to be important (15). We speculate these patterns were not explored as a consequence of the multiscale nature of the data. The cursory analysis demonstrates some of the potential of unsupervised and structured diagnoses of mrCOSTS for multiscale data.

### Mountain Boundary Layer (MoBL).

The MoBL is well known for its multiscale dynamics, especially in stable conditions when processes at the so-called “submeso scales” are important but poorly understood (64–67). The submeso scales are processes with time scales roughly between 10 min to an hour but are difficult to precisely define due to overlapping process scales (67, 68), dynamics occurring across orders of magnitude spatiotemporal scales (67, 69, 70), and nonstationary and translating features, all with fairly limited, and frequently noisy, observations (68). Thus, the multiscale dynamics of the MoBL, especially at the submeso scales, form a fundamental obstacle to the modeling, understanding, and prediction of the atmosphere (66, 67, 70). We draw specific attention to nonmonochromatic waves, which are common submeso scale features, as we lack robust methodological tools for analyzing them (65, 71–74). For this reason, we subjectively characterize the MoBL data as the most difficult example of multiscale dynamics examined in this study.

The MoBL is an illustrative example of the challenges of discovering unknown dynamics from multiscale data. Atmospheric models are developed by defining a grid spacing. The size of the grid spacing dictates which processes are explicitly resolved (those larger than the grid spacing) and those which must be parameterized (those smaller than the grid spacing; refs. 13, 66, 75, and 76). The construction of these parameterizations relies on understanding how processes at a smaller scale create emergent behavior at a larger scale, which has to be informed by data (66, 77, 78). As the grid spacing shrinks, i.e., when seeking to improve the model, new parameterizations have to be derived (13, 79). These improvements require richer, more detailed data, which due to their multiscale nature, come up against the limits of traditional analytical techniques (68). Thus, even though the physical laws of fluid flows are well known, describing the emergent physics across scales remains a fundamental need and challenge, in part because of the multiscale nature of MoBL data.

An observational campaign was conducted in the summer of 2022 in the Inn Valley, Austria for the explicit purpose of addressing these challenges (68). We make use of the coplanar retrieval of horizontal wind speed components, u (east-west component) and v (north–south component), using two horizontally oriented LIDARs with overlapping fields of view (80). These types of data are generally not amenable to objective analytical methods and instead require careful human interpretation. The wind speed components were rotated into a coordinate system better aligned with the Inn Valley axis (roughly southeast to northwest), yielding an across-valley component, $v_{⊥}$ and an along-valley component, $u_{\|}$.

The coplanar observations were set up to observe the outflow of the tributary Weer Valley into the main Inn Valley 60 m above the valley floor. The Inn Valley at this location is approximately 2 km deep and 3 km wide while the tributary Weer Valley is 60 m deep and ≈250 m wide at the height of the LIDAR observations. Thus, the tributary flow is characterized by length scales an order of magnitude smaller than the main valley flow (68). The main valley circulation at night with weak synoptic forcing should be down-valley due to a thermally driven circulation (67, 69) and tributary flows should act to enhance the down-valley component. However, it remains unknown how tributary and main valley flows couple, as previous work found that the mass flux enhancement in a main valley was more than the outflow of the tributary valley alone (69, 81, 82). Additionally, oscillations can be found at the same frequency in both systems (69, 81) but proposed coupling mechanisms could not be verified (82).

As in the neurology example, we performed a small amount of hyperparameter tuning in order to capture low-frequency dynamics which were only apparent after performing an initial mrCOSTS decomposition. From the individual $G_{p}$ we formed three aggregate bands of 141 to 68 min, 52 to 26 min, and 17 to 6 min using Eq. **3** for visual clarity, as these bands captured the major dynamics of the system (all bands are shown in *SI Appendix*, Fig. S3).

**Fig. 4.**
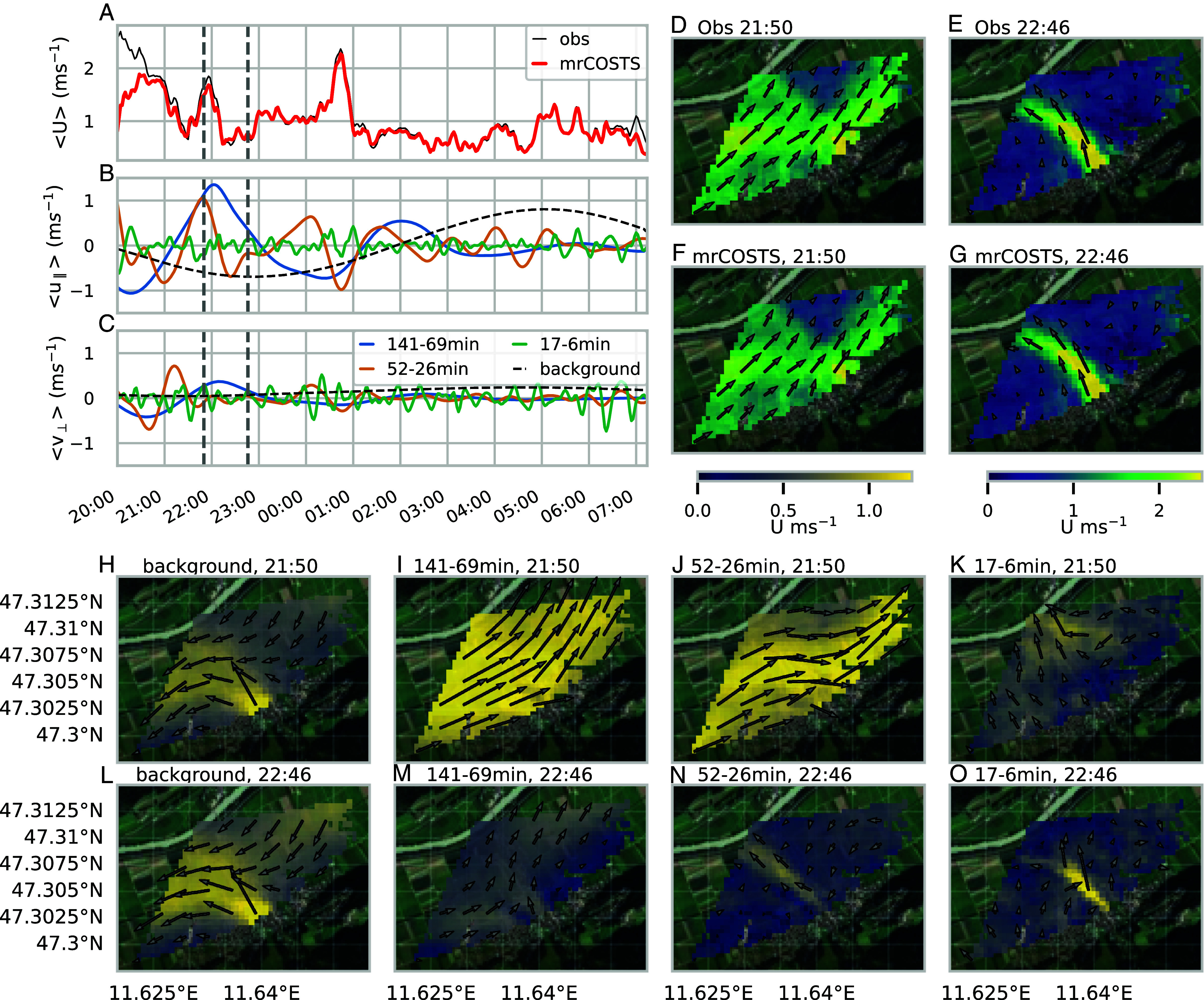
An overview of the mrCOSTS decomposition of the MoBL data highlighting two times of interest; see Movie S2 for all times. These dynamics were found with minimal hyperparameter tuning. The mean horizontal wind speed is $U=\sqrt{u^{2}+v^{2}}$ and <> denotes a spatial mean. (*A*) A comparison of the observed and mrCOSTS reconstruction of the mean wind speed. The time series of the aggregated bands for (*B*) the along-valley wind $\left\langle u_{\|}\right\rangle$, (*C*) the across-valley wind $\left\langle v_{⊥}\right\rangle$ are shown. (*D* and *E*) The coplanar retrieval of wind speed and direction approximately 60 m above the valley floor at two times with (*F* and *G*) the mrCOSTS reconstructions. The two times are indicated as vertical dashed lines in (*A*–*C*). The reconstructed mean wind speed and direction for the background mode and the three aggregated bands are shown for the first (*H*–*K*) and second (*L*–*O*) times of interest. The mrCOSTS decomposition reveals the complex interaction of the background, seiche-like oscillations, and tributary valley in-flow which could not be well-characterized previously. The background image ©2024 CNES/Airbus, Google, Maxar Technologies.

The mrCOSTS decomposition reveals a remarkable oscillation of the wind along the valley axis. These oscillations were most prominent in the 95 and 52 min bands (*SI Appendix*, Fig. S3), which dominate the aggregated band behavior (blue and orange lines in Fig. 4*B*, respectively). The clean and regular oscillations of $u_{\|}$ in the nonaggregated bands (*SI Appendix*, Fig. S3), are suggestive of a standing wave such as a seiche. Seiches are most well-known in enclosed basins in the atmosphere (69, 83), which may potentially allow for cleaner harmonics that can be more easily determined both analytically and visually. The boundary upon which the waves would reflect is unclear but potentially could be related to the bend in the valley system immediately to the east of the study area or from persistent density boundaries caused by the multiple tributary in-flows along the main valley.

Regardless of the physical mechanism, the time scales are consistent with submeso scale oscillations observed in other valley systems (69 and references therein). However, oscillations at these submeso scales have not been previously noted in the Inn Valley, despite the Inn Valley being the subject of six field campaigns (68). We speculate that the lack of characterization of these oscillations is due to their complex multiscale nature. Extracting out these kinds of patterns (i.e., nonmonochromatic waves) from spatially distributed data is a major methodological goal (e.g., refs. 65 and 71, 72, 73, 74). Generally, phenomenon like gravity waves are not even explored on these horizontal scales, in part because of a lack of analytical tools for these kind of nonmonochromatic waves, a constraint easily overcome with mrCOSTS.

Isolating the exact physical mechanism is beyond the scope of this study so we will refer to these oscillations as “seiche-like” for simplicity. Instead, we highlight how mrCOSTS revealed the complex multiscale dynamics of the seiche-like oscillations interacting with other processes of the MoBL using two times of interest ($t_{1}=2,150$ and $t_{2}=2,246$; vertical dashed lines in Fig. 4*A*–*C*) while Movie S2 shows the entire time period. At $t_{1}$ the tributary outflow appears to be limited with a substantial down valley flow in its place (Fig. 4 *D* and *F*). However, mrCOSTS instead suggests a very slowly fluctuating background containing a persistent tributary outflow and main valley flow is overwhelmed by the seiche-like motions described by the aggregated bands (Fig. 4*H* vs. 4 *I* and *J*). The highest frequencies reveal a cross valley oscillation (Fig. 4 *C* and *K*). In other words, the dynamics of the tributary valley in-flow was relatively constant in time (Fig. 4*H*) but the seiche-like oscillations along the valley floor masked this behavior (Fig. 4 *I* and *J*).

For $t_{2}$, the slowly fluctuating background mode is largely unchanged from $t_{1}$ except for a strengthening of the up-valley wind (Fig. 4*L*). The seiche-like oscillations of the largest time scales (Fig. 4*M*) oppose the main valley flow, while the finer time scales (Fig. 4 *N* and *O*) reveal a pulse in the tributary flow, which travels into the main valley over the next time steps (Movie S2). The composite effect is to create the appearance of a strong tributary flow penetrating into a weak main valley. Instead, the interplay between the nearly constant background flow and the seiche-like motions dominates the system, even in periods in which we would have interpreted the tributary in-flow as acting alone such as during $t_{2}$. We would also expect a thermally driven down-valley flow in stable conditions with weak synoptic forcing such as these (69) but the background up-valley flow (Fig. 4 *H* and *L* and negative $u_{\|}$ in Fig. 4*B*) suggests that other processes are relevant. However, background modes from mrCOSTS are not well-resolved and require larger decomposition windows to be described in detail.

Being able to rigorously diagnose these kinds of multiscale processes should enable refined interpretations of these data as well as develop new physical explanations. Characterizing these previously difficult to describe dynamics in a well-studied system like the Inn Valley further underlines the utility offered by a diagnostic like mrCOSTS.

## Discussion

In summary, we have provided a definition for multiscale data for which existing computational methods are inadequate. To answer the challenge of analyzing multiscale data, we provided mrCOSTS, a robust, unsupervised method capable of diagnosing these data with minimal hyperparameter tuning. It adapts the mrDMD framework for the complexities of real multiscale data. The substantial algorithmic and performance changes between mrCOSTS and the original mrDMD algorithm highlight the importance of testing on real data and not just synthetic or simple examples. In exceptionally challenging real-world data considered here, mrCOSTS already shows its value as an unsupervised algorithm by extracting spatiotemporal patterns in well-studied systems that would be otherwise difficult to characterize due to the complexities of the underlying dynamics.

Specifically, we demonstrated mrCOSTS on data from a range of fields ranging from biology (neurology) to climate/weather, which were explicitly chosen for their well-known multiscale dynamics. In the SST example, we diagnosed a high-dimensional signal with nonstationarity properties. The known time dynamics were recovered along with their coherent spatial patterns, a useful result on its own. We additionally identified three time bands outside the normally defined ENSO temporal scales, which were critical for explaining the time series of the ENSO anomaly as well as the extreme behavior of the 2015–2016 ENSO event (Fig. 2). In the neurology example, we deconstructed the LFP signal into coherent spatiotemporal patterns of well-known frequency bands and revealed their complex translating properties (Fig. 3 and Movie S1). In the MoBL example, we characterized dynamics that evaded prior detection in the Inn Valley despite extensive study, revealing a complex interplay of processes at the submeso scales including nonmonochromatic waves (Fig. 4 and *SI Appendix*, Fig. S3 and Movie S2). In each of these cases, either no or exceptionally limited hyperparameter tuning was performed to highlight the unsupervised diagnostic power provided by mrCOSTS. Of particular note, noise did not hinder analysis for any of the systems.

Diagnosing coherent spatiotemporal patterns with mrCOSTS has untapped benefits, as we only demonstrate the general application of the method here. For example, in the SST example, the 11.1 y band describes a spatial pattern that extends well beyond the Niño 3.4 box (Fig. 2*D*), while the 1.4 y band is more spatially confined (Fig. 2*I*). The decomposed spatiotemporal patterns should enable a more robust description of ENSO events compared to the wide range of metrics currently needed to describe the complex spatiotemporal patterns of this system (44, 50–52).

A robust scale separation for multiscale data has been a long sought after goal in a wide range of fields from atmospheric turbulence (65), weather (13), ocean dynamics (84, 85), climate (16–18), neurology (86–88), and others not explored here. Consequently, mrCOSTS has substantial value over other methods for its scale separation properties alone. The bands, $G_{p}$, enable analysis requiring isolated processes describable by specific dynamics (similar to ref. 85). The method has additional potential for enabling the retrieval of physics that emerge from complex interactions across scales. For instance, the interpretable DMD model mrCOSTS is based on can facilitate finding separate models capable of describing the different scales and the coupling between them.

Multiscale data are a major challenge in many disciplines, especially those defining the Grand Challenges in Science and Engineering (2). In order to drive hypothesis testing and process understanding forward, we must be able to discover the patterns and processes within these complex data. Recovering previously unknown patterns and complex scale interactions is a task well-suited to analysis by data-driven modeling. But, the complexities of these data have somewhat blunted the potential of such approaches. Providing a robust, unsupervised method of scale separation and diagnoses moves us substantially closer to the goal of using data to derive the complex, emergent laws of multiscale systems, but does not solve the problem outright. Substantially more work must be conducted to determine how to best use and improve these diagnoses.

## Materials and Methods

### Methods.

The python implementation of mrCOSTS is available as part of the PyDMD package (23) and is one of the major updates comprising version 1.0 of the package. The implementation includes plotting routines, parameter sweeps, in/out operations for off-line analysis of the fitted model, and tutorials on real and toy data. PyDMD includes automated testing, an active user community, and support for a wide range of other DMD models as well, providing a rich framework for analysis. The code for creating Figs. 2–4 is available at https://github.com/klapo/mrCOSTS-PNAS-figures (89).

#### Practical considerations.

All other DMD variants naturally provide an equation-free method for prediction. mrCOSTS does not provide this ability and further the method loses reliability near the edges of the time domain (24). Unlike some decomposition like MRA, mrCOSTS does not perfectly decompose the input signal. This has advantages, for instance, the exclusion of white noise from fitting. However, some dynamics may not be fit at all, so the fit must be carefully evaluated (as in *SI Appendix*, Figs. S1 and S2).

#### Algorithm details.

Separating frequency bands requires transforming ω. Originally in ref. 24, clustering was performed only on $\omega \omega^{*}$ (where we use $\omega^{*}$ to denote the complex conjugate), which includes the contribution of both Re(ω) and Im(ω). For small amplitude modes, the real and imaginary components can alternate between adjacent windows with similar values (e.g., 10+0i vs. 0+10i). The transformation $\omega \omega^{*}$ treats these values as identical, contaminating the frequency band identification. mrCOSTS supports multiple methods of transforming ω for clustering frequency bands: |Im(ω)|, ${\left|Im\left(\omega \right)\right|}^{2}$, and $\log_{10}\left(|Im\left(\omega \right)|\right)$. We find |Im(ω)| is often the appropriate choice of transformation for the local scale separation. For the global frequency band identification, clustering must be performed using the $\log_{10}\left(|Im\left(\omega \right)|\right)$ transformation. Otherwise, the frequency bands, which can span order of magnitudes of scales, cannot be reliably separated.

The DMD model fit to each window is the variable projection optimized DMD (90). We introduce a powerful innovation which enables placing arbitrary constraints on the eigenvalue solutions and is now implemented in PyDMD (23). We found in particular that requiring the real component of the eigenvalues to be small, but not exactly zero, enables robust fitting of data. Most physical systems, while the data may be nonstationary or nonlinear, do not have unconstrained growth or decay and by imposing this eigenvalue constraint we recognize this feature. Without this constraint, or alternatively by imposing Re(ω)≤0, individual windowed DMD fits can be poor, especially when fitting the smaller decomposition levels. See *SI Appendix* for notes for strategies of creating initial guesses of the eigenvalues in each window.

#### Hyperparameters.

In order to objectively select hyperparameters, fits should be evaluated against the reconstruction error and recovery of the power spectra, as in Fig. 3 and *SI Appendix*, Figs. S1 and S2. We explicitly refrained from hyperparameter tuning, except for adding a decomposition level in the neurology and MoBL examples, to demonstrate this point. The most sensitive parameters are the rank of the DMD fit (r), the window size for each decomposition level (w) as well as the number of decomposition levels (N), and the constraints on the eigenvalues. These are the hyperparameters to adjust first when encountering a bad fit.

The number of resolved bands, p, approximately scales with $\frac{r}{2}$. The window size for each decomposition level determines the largest resolvable high-frequency component, e.g., a small window size can fit higher frequency components while a larger window size can fit lower frequency components. Dyadic scaling of the window size is not necessary (see the MoBL for example) and qualitatively similar results can be recovered for varying window sizes. Since strict scaling is not necessary, window sizes can be chosen to target scales of interest. Typically, the smallest window size is the most finicky since the window length needs to be slightly larger than r but setting it to be too large means omitting the finest scale features. Generally, the windowed DMD fit scales closer to the window size and omits the highest frequency components creating a trade-off between a large enough r and a small enough w to capture the highest frequency components. Missing frequencies in $G_{p}$ can usually be solved by increasing r for the relevant decomposition level(s).

The number of frequency bands, both for the global and local separations, can be provided as an a priori expectation or found objectively using k-means clustering from scikit-learn (27) with a hyperparameter sweep. The optimal number of frequency bands is found using the Silhouette score (91), which is well suited for clustering of the flattened ω arrays. Other metrics and clustering algorithms were tested and found to perform substantially worse.

#### Sea surface temperatures.

We use the Hadley Centre Sea Ice and Sea Surface Temperature dataset (92) using the SST data between 30^°^ S and 30^°^ N and 100^°^ W and 120^°^ E. HadISST data (92) are publicly available at https://www.metoffice.gov.uk/hadobs/hadisst/ (93). We calculate SST^∗^ relative to a moving 30 y historical period and smoothed by a rolling 3 mo average (as in ref. 53). When evaluating within the Niño 3.4 box, SST^∗^ is relative to the spatial mean within the box (consistent with ref. 53), while for the entire domain, the anomaly is calculated relative to each grid point individually.

For the decomposition, no hyperparameter tuning was performed (see *SI Appendix* for fit hyperparameters). The total error of the reconstruction over the entire period was 11%, with most of the error located at the subseasonal scales and edge effects (*SI Appendix*, Fig. S1). The fit could be improved by increasing r, especially for the smaller windows, and constraining the eigenvalues to have a small Re(ω). This was not performed as it was unclear what would constitute a small Re(ω) until after the first decomposition was performed and due to the unknown trade-off between r and the window length of the first decomposition level.

#### Neurology.

The neurology data can be accessed through the https://doi.org/10.12751/g-node.f83565 (54, 63, 94). The data for trail 2 of monkey L were quality-controlled, removing points with exceptionally large or small temporal deviations or uncharacteristic jumps, resulting in 7 of the 96 electrodes being discarded and a total of 11 missing points in the spatial map of the electrodes (Fig. 3*D*–*G*). The decomposition was performed from the start of the trial to the end of the reward being dispensed.

The mrCOSTS reconstruction had an error of 21% for the full period and 14% when excluding the edge effects (approximately 0.15 s at the start and end). Most of the error was a consequence of dropping white noise, poorly resolved high frequencies (Fig. 3*D*), and edge effects. See *SI Appendix* for fit hyperparameters.

#### Mountain boundary layer.

The LIDAR coplanar data can be accessed at https://doi.org/10.5281/zenodo.7212801 (68, 95). The coplanar retrieval (80) was conducted using two LIDARS placed on the north valley slope, opposite of the tributary valley in-flow. The LIDAR retrieval yielded only radial velocity relative to the instrument, but using colocated observations, the u and v velocity components were retrieved. A complete observation of the coplanar area, i.e., the data time step, was made every 2 min. Gaps for individual grid points less than 8 min were filled using a linear interpolation in time.

We decomposed a single night from 2,000 on August 12th to 0715 on August 13th. There was an upper level low over the Inn Valley during this time but a small pressure gradient at the height of the Alpine crest, which is the relevant level for forcing the Inn Valley (96). We focused on nighttime in part to diagnose submeso scale processes but also because the data quality degrades substantially during the day.

The mrCOSTS fit had an error of 23 and 26% for the u and v components, respectively. The majority of the error was located at the beginning and end of the decomposition due to the edge effects. The error decreases to 13% and 15% when excluding the first and last 20 min (*SI Appendix*, Fig. S2). The mrCOSTS decomposition recovered the complex temporal dynamics except at time scales smaller than 8 min (*SI Appendix*, Fig. S2 *A* and *B*) as a result of the relatively coarse observation time step, instrument noise, observation gaps, and prioritizing larger scales with the hyperparameters (see *SI Appendix* for fit hyperparameters).

The decomposition accurately recovered u and v low-order statistical moments (*SI Appendix*, Fig. S2 *C*–*F*), demonstrating how mrCOSTS can robustly decompose multiple variables through their shared covariance. As a result, <U> was also accurately reconstructed from the mrCOSTS fit (Fig. 4*A*). The error in the mrCOSTS fit was mostly caused by missing the small time scales, removing white noise, and the edge effects.

## Supplementary Material

Appendix 01 (PDF)

Movie S1. Movie version of Fig. 3 of the main text, but showing all bands and times from the neurology case study. The (a) observed and (b) reconstructed LFP data by electrode and time as well as (c) the specific frequency bands are shown. The vertical dashed line indicates the time of the movie visually, which is also indicated in the top left corner of (b). (d-g) Each frequency band is mapped to the x-y location of the electrodes with the spatial patterns displayed using z-scores to allow plotting them on a uniform color scale.

Movie S2. Movie version of Fig. 4 of the main text, but showing all of the aggregated bands and times from the MoBL case study. The contributions of the aggregated bands to the (a) spatial mean of the horizontal wind as well as (b) along- and (c) across-valley wind components. The vertical dashed line indicates the time of the movie visually in (a-c). The time is also displayed in the upper left hand corner of (d). The (d) data and (e) mrCOSTS reconstruction as well as the (f-h) reconstructions of the aggregated bands for each time step are shown. The color scale and arrow length are scaled separately between (d-e) and (f-h). Background image ©2024 CNES/Airbus, Google, Maxar Technologies.

## Data Availability

Code has been deposited in GitHub (https://github.com/klapo/mrCOSTS-PNAS-figures) (89). Previously published data were used for this work (https://10.1029/2002JD002670; https://doi.org/10.12751/g-node.f83565; and https://doi.org/10.5281/zenodo.7212801) (93, 94, 95).
