# Supplementary material for: A method for unsupervised learning of coherent spatiotemporal patterns in multiscale data
Source: Proc Natl Acad Sci U S A. 2025 Feb 14;122(7):e2415786122. doi: 10.1073/pnas.2415786122 (PMC11848389; doi:10.1073/pnas.2415786122)
Supplement: Supplementary file 1 — Appendix 01 (PDF) [file pnas.2415786122.sapp.pdf]

Supporting Information for

Unsupervised multi-scale diagnostics

Karl Lapo, Sara M. Ichinaga, J. Nathan Kutz

Karl Lapo.
E-mail: karl-eric.lapo@uibk.ac.at

This PDF file includes:

Supporting text
Figs. S1 to S3
Table S1
Legends for Movies S1 to S2

Other supporting materials for this manuscript include the following:

Movies S1 to S2

Supporting Information Text

Multi-scale data and typical evaluation methods. We repeat the definition of multi-scale data from the main text verbatim here for ease of reference. We define multi-scale dynamics as those characterized by a combination of at least two of the following properties: being multivariate (i.e. acting along multiple dimensions simultaneously), containing process scales across orders of magnitude, being non-stationary, or having invariances such as translation and rotation. Additionally, these data often contain noise or uncertainty, i.e. from instrument error.

Initializing eigenvalues for solver. We note strategies for finding initial eigenvalues for the solver: resetting the initial value for each window, using the previously found initial value, and using a constant initial guess. Resetting the initial values means that each window is independently initialized. This strategy leads to better overall fits across all windows at the cost of computation time. The next strategy involves using the eigenvalues of the previous window as the initial guess for the current window. This strategy can fail for data without well-separated frequency bands, as is commonly the case for real multi-scale data. It is also possible to use a fixed set of initial eigenvalues for all windows works for systems with regular oscillators common in toy model data but is not generally recommended.

Fit Hyperparameters.

Sea Surface Temperature. To fit the Sea Surface Temperature data, we specified the rank of the decomposition for each level (r=8), decomposition windows of [16, 32, 64, 128, 256, 512, 1024] (dyadic scaling) and a slide of approximately 10% between windows. The initial window length was selected to be larger than the decomposition rank but short enough that seasonal and subseasonal modes could be identified.

Neurolology. To fit the neurology data, the decomposition window sizes were [50, 100, 250, 500, 1000, 2000] microseconds with a slide of 10% of the window length. Each window length was fit using $r = 8$ and we forced each level to find 4 frequency bands for the local scale separation and objectively found the number of global bands using the built-in hyperparameter sweep from PyDMD (i.e., p was not set *a priori*).

Mountain Boundary Layer. To fit the Mountain Boundary Layer data, the window lengths were [24, 48, 96, 192, 360, 480] minutes (the longest window was 8 hours in length) with the slide between windows being 10% of the window length. Each window length was fit using $r = 8$ and we forced each level to find 4 frequency bands for the local scale separation and objectively found the number of global bands using the built-in hyperparameter sweep from PyDMD (i.e., p was not set *a priori*).

Evaluating mrCOSTS fits. The evaluation of each of the case studies considered in the main text is highlighted in more detail here. We make use of the power spectral density to highlight which scales were fit and demonstrate the overall fidelity of the fits using lower order statistical moments. An equivalent evaluation of the neurology LFP data is included in the main text. The complex translating and non-stationary behavior of the neurology and MoBL examples are additionally highlighted in the SI movies.

Table S1. A summary of the performance of various unsupervised methods for features defining multi-scale data.

Method	Short Description	Non-stationary	multi-variate	process across scales	invariances	handles noise
mrCOSTS	Sliding window decomposition using DMD into bands of coherent spatial modes which share a narrow range of temporal dynamics	Yes	Yes	Yes	Yes	Yes
PCA (and other modal analysis)	Decomposes into modes of dominant variability	Partially[1]	Partially[1]	No[2]	No[3]	Yes
CWT, Periodograms (and other TFA *with windowing*)	Discover frequencies present in time series *with windowing*	Yes	Partially[4]	Yes	No[3]	Yes
DMD (without windowing)	Find spatial patterns which share a single set of time dynamics across the entire time domain	No[5]	Yes	Yes	No[3]	Partially[6]
Power spectra, FFT, and other TFA *without windowing*	Decompose the time series into its constituent sinusoidal frequencies *without windowing*	Partially[7]	Partially[4]	Yes	No[4]	Yes
non-stationary DMD	Windowed DMD which allows mode frequencies to slowly evolve between windows, but only uses a single window size.	Yes	Yes	No[8]	No[3]	Yes
Physics-informed DMD[9]	Imposes constraints on DMD solutions such as conservation to better model specific known physics.	No	Yes	Yes	Yes	Yes

See main body text for supporting citations. In each case the performance refers to "off the shelf" application of the method without human intervention. TFA refers to Time-frequency analysis, PCA is Principal Component Analysis, FFT is Fast Fourier Transform, CWT is Continuous Wavelet Transform.

[1] Breaks correlation between spatial modes and time dynamics.
[2] Generally require a large number of modes to represent time scales across orders of magnitudes.
[3] Cannot diagnose invariances such as translation or rotation
[4] Co-spectra can diagnose shared dynamics between variables but only operates only along a single dimension at a time.
[5] Temporal dynamics can only exponentially grow or decay, limiting ability to diagnose non-stationary processes.
[6] Depends on the algorithm.
[7] Cannot determine when a frequency was present, only that it contributes to the signal.
[8] Only considers a decomposition at a small range of temporal scales
[9] Is not unsupervised. Requires knowledge of underlying physics.

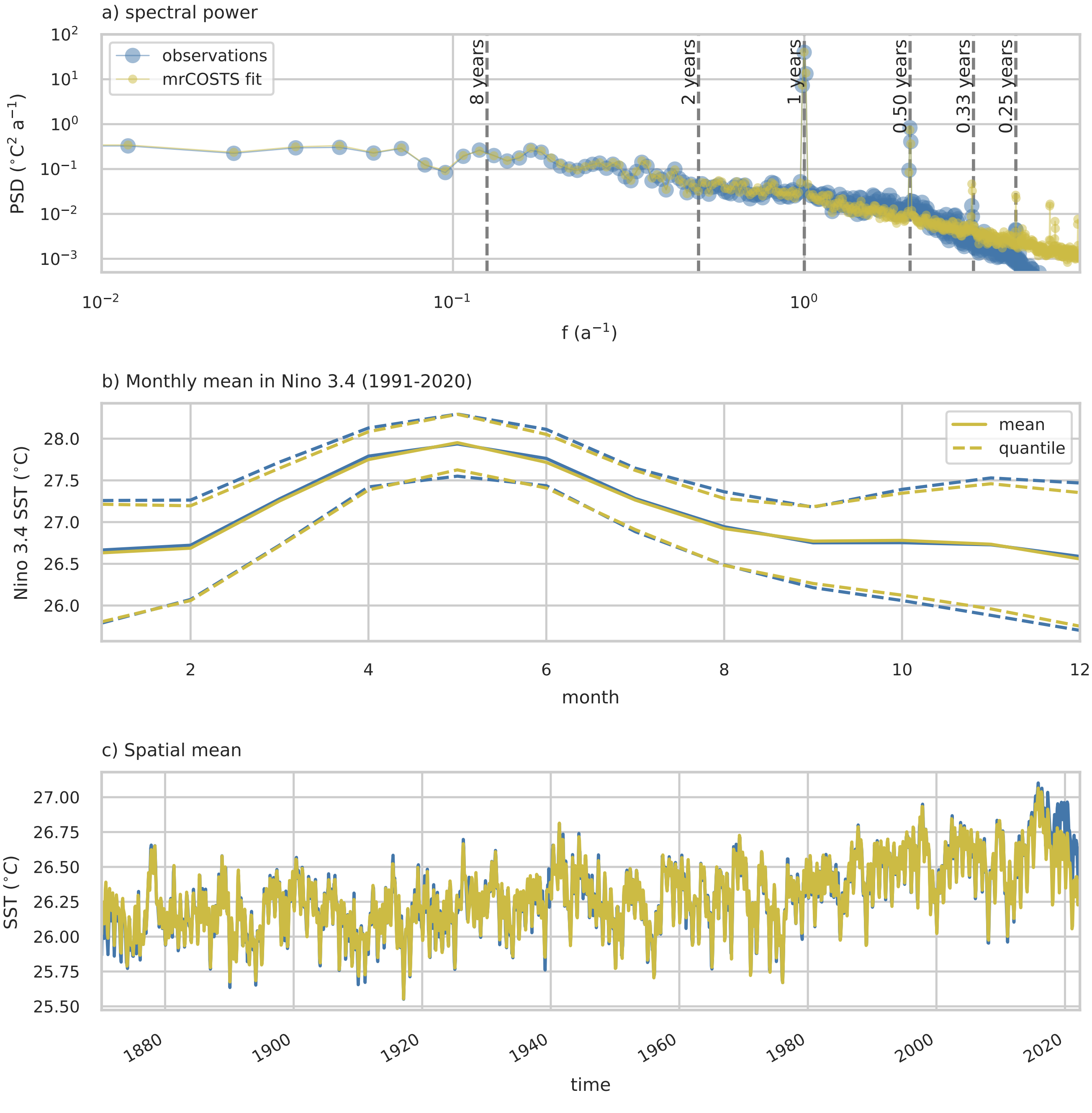

Fig. S1. Evaluation of the SST decomposition demonstrating that mrCOSTS recovered the expected dynamics. (a) The power spectral density of the observed SST (blue) and mrCOSTS reconstruction (yellow). Even though mrCOSTS misses some of the sub-annual power it accurately recovers the annual and seasonal harmonics, which are indicated using vertical dashed lines. The typical ENSO time period (2-8 years) are also indicated to demonstrate how mrCOSTS accurately recovers the desired dynamics in this range of time scales. (b) The composite monthly means as well as the variance of SST in the Nino 3.4 box are accurately recovered (colors same as in a). Finally, (c) the time series of the SST spatial mean of the entire region is shown. The decomposition has larger errors at the beginning and end of the window but accurately recovers the time dynamics of the signal.

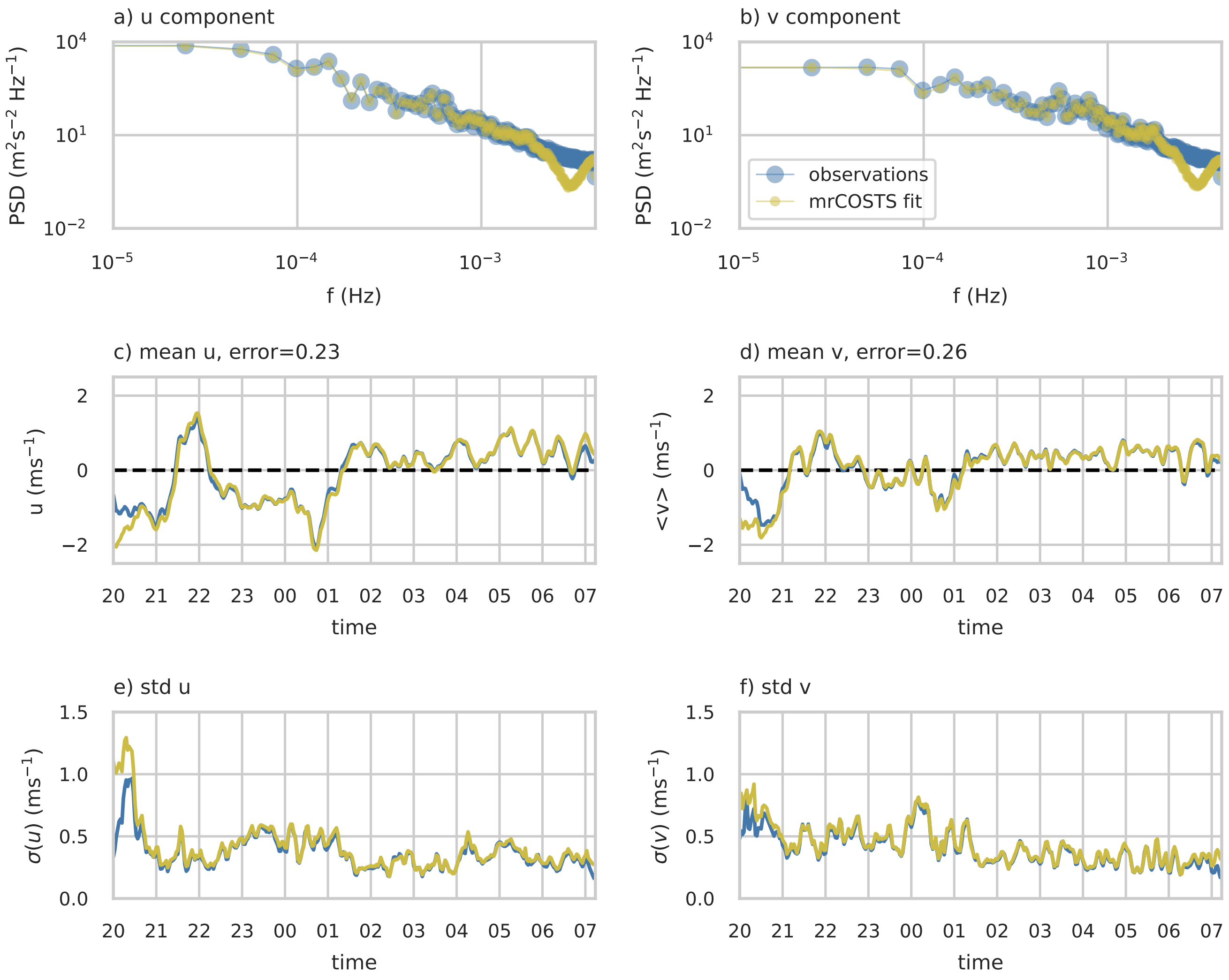

Fig. S2. Evaluation of the MoBL decomposition demonstrating that mrCOSTS recovered the expected dynamics. (a) The power spectral density of the (a) u and (b) v components of the flow. Although mrCOSTS misses some of the power below time scales of $\approx$ 8 minutes, it accurately recovers all longer time scales. The spatial mean of (c) u and (d) v and the standard deviation of (e) u and (f) v for both the observations and the decomposition are plotted.

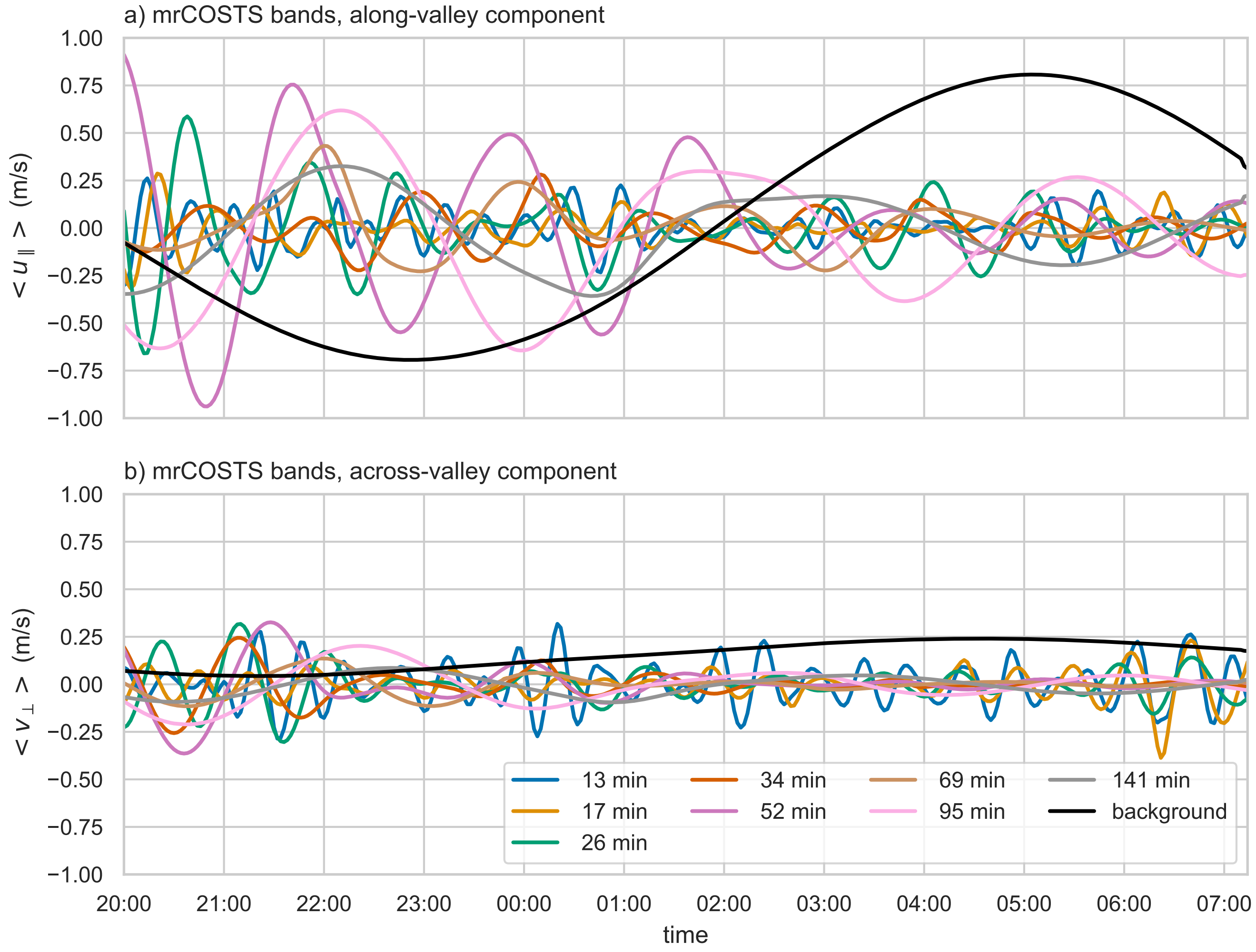

Fig. S3. Demonstrating the behavior of the individual, non-aggregated $\mathcal{G}_p$ highlighting the clean oscillations at the 52 min and 95 min time scales. The area-averaged (a) along and (b) cross valley wind components for the individual bands.

Movie S1. Movie version of Fig. 3 of the main text, but showing all bands and times from the neurology case study. The (a) observed and (b) reconstructed LFP data by electrode and time as well as (c) the specific frequency bands are shown. The vertical dashed line indicates the time of the movie visually, which is also indicated in the top left corner of (b). (d-g) Each frequency band is mapped to the x-y location of the electrodes with the spatial patterns displayed using z-scores to allow plotting them on a uniform color scale.

Movie S2. Movie version of Fig. 4 of the main text, but showing all of the aggregated bands and times from the MoBL case study. The contributions of the aggregated bands to the (a) spatial mean of the horizontal wind as well as (b) along- and (c) across-valley wind components. The vertical dashed line indicates the time of the movie visually in (a-c). The time is also displayed in the upper left hand corner of (d). The (d) data and (e) mrCOSTS reconstruction as well as the (f-h) reconstructions of the aggregated bands for each time step are shown. The color scale and arrow length are scaled separately between (d-e) and (f-h). Background image ©2024 CNES/Airbus, Google, Maxar Technologies.
